# Supplementary material for: Genome and Tissue‐Specific Transcriptome of the Tropical Milkweed ( Asclepias curassavica )
Source: Plant Direct. 2025 Mar 18;9(3):e70031. doi: 10.1002/pld3.70031 (PMC11914377; doi:10.1002/pld3.70031)
Supplement: Supplementary file 1 — Data S1 Peer Review [file PLD3-9-e70031-s008.pdf]

Plant Direct
Open Access

**Genome and tissue-specific transcriptome of the tropical milkweed (Asclepias curassavica)**

**Honglin Feng1,5 , Jing Zhang1 , Adrian F. Powell1 , Gretta L. Buttelmann4 , Lily Yang1 , Ethan Yan1 , Fumin Wang1 , Steven B. Broyles2 , Georg Jander1* , and Susan R. Strickler3,4***

**Decision Letter Round 1:**

February 25, 2024

Georg Jander
Boyce Thompson Institute
533 Tower Rd
Ithaca, New York 14853

RE: Genome and tissue-specific transcriptome of the tropical milkweed (Asclepias curassavica)

Dear Dr. Jander and Dr. Strickler:

Thank you for submitting to Plant Direct. All required reviews have been returned and we have now finished our evaluation of your manuscript. In light of the reviewers' and editor's comments, further revisions are needed before the paper can be accepted for publication in Plant Direct.

Please note that these revisions are minor and primarily involve clarifications and reformatting. No new experiments are required.

Please view the editors' and reviewers' comments below and use their suggestions as a guide while you work on your revision.

When uploading the revised version of this article, please be sure to include the following:

-A word document that contains your response to the reviewers. You should respond to each reviewer comment and note the changes made to the manuscript. If you do not agree with a reviewer's comment and choose not to make a suggested revision, please explain why. Please try to provide as complete an answer as possible to each reviewer's criticisms.
-A tracked changes document with each change highlighted
- A clean version of the latest version of the manuscript

Plant Direct
Open Access

To upload your revision, please click the link below.
https://plantdirect.msubmit.net/cgi-bin/main.plex?el=A4Lr5ft1A4BIW5I7A9ftdZuWrVuLzslWWxk7UWqP7gZ

In order to provide as timely a service as possible, we ask that your revision is resubmitted within three months after receipt of this request. If an extension is needed, please send a request, along with a brief explanation, to the editorial office at plantdirect@wiley.com .

Please note that, in addition to publishing reviewer comments, the author's responses to review comments will also be published alongside the final version of the paper. If you would not like the author's responses to be published, please contact the editorial office at plantdirect@wiley.com .

Thank you very much for giving us an opportunity to review your work. I look forward to receiving the next version.

Sincerely,

Gaurav Moghe

Hsou-min Li

Editor, Plant Direct

-------------------------------------------------------------------------- Editor comments:
* Reviewer 1 has provided relevant feedback about FIg. 1, which can be addressed.

* Fig. S2: Is it normal to have so many HiC contacts between chromosomes?

* Fig. 5: Please provide a timescale of divergence between species e.g. using timetree of life. Also, spelling mistake in caption

* In the gene family analysis, it would be good to provide a short explanation of what the lambda rate is.

* I could not find the discussed genes (five JAZ, LOX, ACOX1) in Fig. 8. Would be good to highlight these genes e.g. with an arrow. The JAZs are spread out and I could not find ACOX1. Similar concerns were also raised by Reviewer 1.

Plant Direct
Open Access

* Please respond to Reviewer #1's comments about the cardenolide level changes. Additional experiments or inclusion of additional data are encouraged but not required.

* Reviewer #2's comments about the significance of some analyses and the disparity between flow cytometry-kmer estimates need to be addressed.

* Discussion says the two Asclepias chromosomes are collinear, however, there are some inversions and chromosome fusions/breakage shown in Fig. 3 (chr 1, 10, 11)

------------------------------------------------------------------------------ Reviewer comments:
Reviewer #1:

Feng et al. describes the "Genome and tissue-specific transcriptome of the tropical milkweed (Asclepias curassavica)", which is indeed substantial producer of cardenolides, also known as cardiac glycosides. The genomic studies presented here will be quite useful to study the biosynthetic pathways of cardiac glycosides in future. Overall, the manuscript is well written. I have very specific comments that can be useful to improve the manuscript:

Introduction: Authors mentioned that the genus Ascelpias are known for their production of cardiac glycosides by referring Figure 1A, which showed structure of voruscharin, the most abundant cardiac glycoside. I think showing one structure is not enough to support this statement. In addition, voruscharin is never mentioned somewhere in the text of manuscript. In which tissue it is accumulated? Authors generated the transcriptome data from several tissues; I did not find the cardiac glycosides levels, at least for major ones accumulating in these different tissues. Did I miss this in the manuscript? This is very relevant information for metabolite-gene correlation studies for pathway gene discovery. Also small schematic showing cardiac glycoside biosynthetic pathway (not necessarily all steps) would be useful to understand the context easily.

Please pay attention for typos and proper use of italics wherever necessary, for example result subheading: Evolution of the A. curassavica genome etc.

Was there any change in cardiac glycosides in different tissues after methyl jasmonate treatment? Is leaf tissue the site of biosynthesis and/or accumulation for cardiac glycosides?

Figure 8 is not providing the key information, as there are several hundred genes, no highlight, no grouping according to cellular processes etc. I had to zoom the figure and look gene by gene wherever figure 8 was motioned in the manuscript. At least, for the genes described in the results text, show it in color or highlight them with shapes etc. Another option is to modify the figure, show only relevant genes supporting the result text so it will be easy to understand. Move full version of figure 8 to supplementary while small informative version can be as main figure.

Figure S4 is not making any sense to the manuscript. Authors mentioned that there is likely a homolog in A. curassavica (Figures S4 and S5). How the multiple sequence alignment can support this? It would be

appropriate to show percent homology identity matrix for these proteins. In addition, phylogenetic analysis of CYP87A4 did not include Digitalis or Erysimum orthologues, which produce cardiac glycosides. Do other plant species shown in phylogeny produce cardiac glycosides? What is the interpretation from this phylogeny? Figure S5 does not support the authors claim that “there is likely a homolog in A. curassavica (Figures S4 and S5).

Table S3: color scheme used is not clear. At one instance, it was used to show cytochrome 450 and in another instance, it was shown to represent for example, jasmonic acid biosynthesis. Use either metabolic/cellular process specific color scheme or genes specific color scheme.

I am quite surprised that Authors did not emphasize on the expression pattern of known genes in cardiac glycoside biosynthesis. CYP87A4, 3bhsd, P5BR?? No mention about how many gene copies or isoforms exist for these genes? Their expression patterns across different tissues? Again no metabolic data shown for any tissues. This is useful information to describe in the manuscript for a milkweed category plant, which is ecological model and substantial producer of cardiac glycosides

Reviewer #2:

The authors present a high quality genome assembly along with associated tissue specific transcriptomes for Asclepias curassavica. The assembly appears to be highly complete ("chromosome level") based on comparison with existing genomic resources in Asclepias as well as BUSCO scores. RNA seq data was generated for plants with and without methyl jasmonate treatment and tested for differential expression of transcripts relative to treatment and tissue type. This paper is well written, and all of the methods are well established. The assembly and associated transcriptomic data represent a significant resource for future studies on cardiac glycoside biosynthesis and evolutionary biology of Apocyanceae. I am happy to recommend this paper for publication pending a couple of clarifications.

First, not being very familiar with cardiac glycoside biosynthesis/secondary metabolism, I'm not sure I understand the significance of treatment with methyl jasmonate. A brief statement in the introduction explaining/justifying this treatment in relation to the stated interest in cardiac glycoside biosynthesis would help make this paper of broader interest.

In the results you list several genes/gene families (e.g. GGT1, GGPS, AMT1-2) that are up/down regulated across all tissues in response to MeJa treatment. However, you don't state why these specific genes are significant relative to the many other genes that are found to be differentially expressed across all tissue types. Why mention these specific genes?

The wide range of genome size estimates is a bit odd, though not entirely surprising due to the limitations of flow cytometry and kmer analysis. Is there any specific explanation for this in Asclepias? Given the high sequencing depth with long and short reads, scaffolding with HiC, and synteny there is

little question that this assembly is highly complete (but also see my question about the comparison with the A. syriaca genome above). However, this stood out to me as something that might merit more consideration.

**Decision Letter Round 2:**

May 13, 2024

Georg Jander
Boyce Thompson Institute
533 Tower Rd
Ithaca, New York 14853

RE: Genome and tissue-specific transcriptome of the tropical milkweed (Asclepias curassavica)

Dear Dr. Jander:

Thank you for submitting to Plant Direct. All required reviews have been returned and we have now finished our evaluation of your manuscript. In light of the reviewers' and editor's comments, further revisions are needed before the paper can be accepted for publication in Plant Direct.

Please view the editors' and reviewers' comments below and use their suggestions as a guide while you work on your revision.

When uploading the revised version of this article, please be sure to include the following:

-A word document that contains your response to the reviewers. You should respond to each reviewer comment and note the changes made to the manuscript. If you do not agree with a reviewer's comment and choose not to make a suggested revision, please explain why. Please try to provide as complete an answer as possible to each reviewer's criticisms.
-A tracked changes document with each change highlighted
- A clean version of the latest version of the manuscript

Thank you for addressing all prior reviewer and editor comments. Due to unavailability of one of the original reviewers, another reviewer was solicited for this revision. They have made some additional comments about the genome assembly. Please see my additional comments regarding individual reviewer comments

To upload your revision, please click the link below.
https://plantdirect.msubmit.net/cgi-

bin/main.plex?el=A4Lr3ft3B5BIW5I7A9ftdZuWrVuLzslWWxk7UWqP7gZ

In order to provide as timely a service as possible, we ask that your revision is resubmitted within three months after receipt of this request. If an extension is needed, please send a request, along with a brief explanation, to the editorial office at plantdirect@wiley.com .

Please note that, in addition to publishing reviewer comments, the author's responses to review comments will also be published alongside the final version of the paper. If you would not like the author's responses to be published, please contact the editorial office at plantdirect@wiley.com .

Thank you very much for giving us an opportunity to review your work. I look forward to receiving the next version.

Sincerely,

Gaurav Moghe

Editor, Plant Direct

------------------------------------------------------------------------------ Editor comments:
Thank you for addressing all prior reviewer and editor comments. Due to unavailability of one of the original reviewers, another reviewer was solicited for this revision. They have made some additional comments about the genome assembly. Please see my additional comments regarding individual reviewer comments. Only 1 analysis may be needed, and other comments simply require more clarification.

------------------------------------------------------------------------------ Reviewer comments:
Reviewer #2:

The authors have addressed all comments and questions about the manuscript to my satisfaction. I am happy to recommend it for publication.

Reviewer #3:

The authors worked on Asclepias curassavica, a tropical milkweed's de novo genome assembly, annotation, and tissue-specific transcriptomes of treated (with methyl jasmonate) and control to induce

biotic defense pathways known to this plant. Although the technical methods (sequencing, assembly, annotation, synteny, gene-centric phylogeny, and DEG experiment and analyses) were scientifically sound, there were a few places where alternative computational methods could have clarified the inconsistencies.

Genome assembly and scaffolding review:
For genome size discrepancies between flow cytometry (639Mbp), k-mer frequency (161Mbp) and assembly (241Mbp), one could do a K-mer frequency analysis of different sizes (other than k= 21) to verify the large heterozygous peak and heterozygosity of 0.2%. Also, demonstrating genome assemblers yielding a similar assembly size may validate the assembly size by MaSuRCA.
****The drastic genome size differences are a serious discrepancy in my opinion, so a varying kmer frequency analysis is a valid suggestion to incorporate. Also, the Lewis paper describes the 2C genome size, while the genome sizes reported here may be 1C.

Since there are multiple mappings of the contact Hi-C map, stating the raw pre- and post-scaffolded assembly metrics, including how many gaps were filled vs joined creating the 233Mbp genome assembly (97% of 241Mbp contig assembly length) and if any of the 851 contigs unscaffolded would support the map. Are there any telomeric and/or centromeric sequences present in this assembly to show validity of the scaffolding (since)

Clearer synteny/gaps between 317Mbp of A. syriaca and the 241Mbp presented A. curassavica genome; i.e a large contracted area between the genomes or is the contracted areas dispersed evenly along the chromosomes

Figure 3: some axes have scale 0 to ~25Mbp and some do not, which make it hard for the reader to gauge synteny between the two genomes. It is puzzling that chromosome 1 seems to be contracted in the middle compared to the reference genome?

Figure S3: Feature density along the Asclepias curassavica pseudomolecules.
What do the colors mean? Seems bizarre that there is high gene density in the middle of chromosome 1 (usually gene density is located at the distal arms of a chromosome like it in in chromosome 1 of A. syriaca)?

Gene and expression analysis review:
For the CYP87A gene family, are there any contractions or expansions in this gene family (since the CAFÉ analysis was done)
**** No additional experiments are needed, only interpretation of results if available.

SMO and SS1 were pointed out to be involved in cardiac glycoside biosynthesis, however SMO seems to be downregulated in all tissues and SS1 is slightly upregulated across all tissues. Is there a hypothesis on why the biosynthetic genes are not upregulated in any tissue?

I am not familiar with the pathway of interest, however, a clearer understanding of why certain genes of this pathway was looked at but not others could help readers also not familiar with the pathway (i.e. https://www.nature.com/articles/srep34464)

Miscellaneous:
Bud and flower are two of the tissue types, could you place add a development time-scale to this?

**Decision Letter Round 3:**

November 11, 2024
Georg Jander
Boyce Thompson Institute for Plant Research
533 Tower Rd
Ithaca, New York 14853

MSID: 2024-01420R2
MS TITLE: Genome and tissue-specific transcriptome of the tropical milkweed (Asclepias curassavica)

Dear Dr. Georg Jander:

I am pleased to inform you that your manuscript "Genome and tissue-specific transcriptome of the tropical milkweed (Asclepias curassavica)" has been accepted for publication in Plant Direct.

Your article will appear online in the next available issue of Plant Direct. To ensure your article gets published as quickly as possible, please pay attention to the steps detailed below. We have found that most of the delays happen at this stage, especially at the payment stage, so please respond as quickly as possible when prompted.

License Agreement: Once your article has been accepted it will move to Production and undergo admin and file checking - you may receive an email with any queries we have at this stage. When all required items are received by the publisher and queries resolved, the corresponding author will receive an email from Wiley's Author Services system which will ask them to log on at https://authorservices.wiley.com/bauthor and will present them with the appropriate license for completion. Your article cannot be published until both the signed license agreement and payment of the article fee have been received.

Payment of the Open Access Article Publication Fee: All articles published in Plant Direct are fully open access: immediately and freely available to read, download and share. Plant Direct charges a publication fee to cover the publication costs. The corresponding author for this manuscript should have already

received a quote from the payments team (cs-openaccess@wiley.com) with the estimated article publication fee; please email cs-openaccess@wiley.com if this has not been received. The corresponding author should log on to the Wiley Author Services site, where the publication fee can be paid by credit card or an invoice. Pro Forma can also be requested. Payment of the publication charge must be received before the article will be published online.

Proofs: You will have the opportunity to look over your paper once more when you receive the author proofs for your article. The proofs will be with you in approximately two weeks. Please note that, in addition to publishing reviewer comments, the author's responses to review comments will also be published alongside the final version of the paper. If you would not like the author's responses to be published, please contact the editorial office at plantdirect@wiley.com .

Promotion of your article: You can help your research get the attention it deserves! Check out Wiley's free Promotion Guide for best-practice recommendations for promoting your work at www.wileyauthors.com/eeo/guide. And learn more about Wiley Editing Services which offers professional video, design, and writing services to create shareable video abstracts, infographics, conference posters, lay summaries, and research news stories for your research at www.wileyauthors.com/eeo/promotion.

Thank you again for your contribution to Plant Direct. If you have any questions, feel free to contact the editorial office at plantdirect@wiley.com .

Sincerely,

Gaurav Moghe

Editor, Plant Direct

-------------------------------------------------------------------------- Editor comments
Please ensure that the BioProject PRJNA1055959 is made available at the earliest. It currently does not show up on NCBI.

-------------------------------------------------------------------------- Reviewer comments:
Reviewer #3:

Thank you for the clarifications and alterations!

**Response to reviewer Round 1:**

Plant Direct
Open Access

In response to comments from the editor and two reviewers, we have revised and improved our manuscript. Specific changes that we have made are detailed below. Text additions to the manuscript file are marked in red in the resubmitted manuscript.

--------------------------------------------------------------------------------------------------------------------------

Editor comments:

* Reviewer 1 has provided relevant feedback about FIg. 1, which can be addressed.
>>We have added additional references and text in the introduction to support the statement that cardiac glycosides are characteristic defensive metabolites in *A. curassavica.*

* Fig. S2: Is it normal to have so many HiC contacts between chromosomes?
>>The between-chromosome contacts are likely due to repetitive elements or transposons. These patterns are common to plant genome assemblies.

* Fig. 5: Please provide a timescale of divergence between species e.g. using timetree of life. Also, spelling mistake in caption
>>We have added this information in the revised manuscript using treePL. We could not use Timetree of Life because it does not contain all of the taxa used in the analysis.

* In the gene family analysis, it would be good to provide a short explanation of what the lambda rate is.
>>We have included the definition in the revised manuscript at the first mention of the lambda rate (in the methods section).

* I could not find the discussed genes (five JAZ, LOX, ACOX1) in Fig. 8. Would be good to highlight these genes e.g. with an arrow. The JAZs are spread out and I could not find ACOX1. Similar concerns were also raised by Reviewer 1.
>>In the revised manuscript, we have added an additional figure highlighting expression of the genes that are discussed in the text.

* Please respond to Reviewer #1's comments about the cardenolide level changes. Additional experiments or inclusion of additional data are encouraged but not required.
>>The purpose of this manuscript was the presentation of the genome as a tool for future milkweed research. We did not compare gene expression and cardiac glycoside content. That will be the subject of future research by us and others.

* Reviewer #2's comments about the significance of some analyses and the disparity between flow cytometry-kmer estimates need to be addressed.
>>As detailed below, we have addressed the Reviewer 2 comments.

* Discussion says the two Asclepias chromosomes are collinear, however, there are some inversions and chromosome fusions/breakage shown in Fig. 3 (chr 1, 10, 11)
>>We have changed this to say that the genomes are "mostly collinear" and point out some of the places where they are not collinear.

---------------------------------------------------------------------------- Reviewer comments:
Reviewer #1:

Feng et al. describes the "Genome and tissue-specific transcriptome of the tropical milkweed (Asclepias curassavica)", which is indeed substantial producer of cardenolides, also known as cardiac glycosides. The genomic studies presented here will be quite useful to study the biosynthetic pathways of cardiac glycosides in future. Overall, the manuscript is well written. I have very specific comments that can be useful to improve the manuscript:

Introduction: Authors mentioned that the genus *Ascelpias* are known for their production of cardiac glycosides by referring Figure 1A, which showed structure of voruscharin, the most abundant cardiac glycoside. I think showing one structure is not enough to support this statement.
>>In the revised manuscript, we have added additional references and text in the introduction to support our statement that the genus *Asclepias* is known for the biosynthesis of cardiac glycosides. As we did not measure cardiac glycosides in this research, we do not think that an extensive discussion of the diversity of cardiac glycosides in genus *Asclepias* is warranted.

In addition, voruscharin is never mentioned somewhere in the text of manuscript. In which tissue it is accumulated?
>>In the introduction, we provide references and more information about the location of voruscharin and other cardiac glycosides in milkweed. We did not measure voruscharin for the research described in this manuscript, we do not know the relative accumulation in different tissue types.

Authors generated the transcriptome data from several tissues; I did not find the cardiac glycosides levels, at least for major ones accumulating in these different tissues. Did I miss this in the manuscript? This is very relevant information for metabolite-gene correlation studies for pathway gene discovery.
>>We did not measure cardiac glycoside levels for this manuscript. The purpose was to assemble a genome that will enable researchers to conduct such gene expression/metabolite correlations in the future. It is certainly something that we and others plan to do in the future.

Also small schematic showing cardiac glycoside biosynthetic pathway (not necessarily all steps) would be useful to understand the context easily.
>> Unlike in the case of *Digitalis* and *Erysimum*, little or nothing is known about the cardiac glycoside biosynthesis pathway in milkweed. The most closely related species with an identified cardiac glycoside biosynthesis gene is *Calotropis procera* (Kunert et al 2023, Nature Plants, DOI: 10.1038/s41477-023-01515-9). Like *Asclepias*, the genus *Calotropis* is in the Apocynaceae. We show this enzymatic step in Figure 9A and identify a likely *A. curassavica* homolog of the *C. procera* protein.

Please pay attention for typos and proper use of italics wherever necessary, for example result subheading: Evolution of the A. curassavica genome etc.
>>In the revised manuscript, we have corrected typos, including the use of italics. In the case of the subheading "*Evolution of the* A. curassavica *genome*", the lack of italics for the species name is correct. The general rule is that, if the surrounding text is italicized for other reasons, then the genus and species names are not italicized.

Was there any change in cardiac glycosides in different tissues after methyl jasmonate treatment? Is leaf tissue the site of biosynthesis and/or accumulation for cardiac glycosides?
>>We did not measure cardiac glycoside biosynthesis for this project. So far, the site of cardiac glycoside biosynthesis in milkweed is not known. Cardiac glycosides are found in the latex which makes it likely to move between different plant parts. Figure 9C suggests that the first step of cardiac glycoside biosynthesis in *A. curassavica* may occur in the stems and roots.

Figure 8 is not providing the key information, as there are several hundred genes, no highlight, no grouping according to cellular processes etc. I had to zoom the figure and look gene by gene wherever figure 8 was motioned in the manuscript.
>>Figure 8 we intend to show the overall pattern of gene induction and repression across tissue types. The genes are grouped according to the similarity of their expression patterns across different tissue types. In the revised manuscript, we show a subset of the genes in Figure 8, with the complete figure moved to supplementary material (Figure S4). Table S3 shows the raw fold-change data underlying the heatmap shown in Figure 8. Additionally, all raw gene expression data are included in Supplemental Dataset S1

At least, for the
genes described in the results text, show it in color or highlight them with shapes etc. Another option is to modify the figure, show only relevant genes supporting the result text so it will be easy to understand. Move full version of figure 8 to supplementary while small informative version can be as main figure.
>>As requested, we have included an additional figure specifically showing the expression patterns of the genes that are mentioned in the text in a more visible manner (Figure 8). The previous Figure 8 has been moved to the supplementary material (Figure S4).

Figure S4 is not making any sense to the manuscript. Authors mentioned that there is likely a homolog in *A. curassavica* (Figures S4 and S5). How the multiple sequence alignment can support this? It would be appropriate to show percent homology identity matrix for these proteins. In addition, phylogenetic analysis of CYP87A4 did not include *Digitalis* or *Erysimum* orthologues, which produce cardiac glycosides. Do other plant species shown in phylogeny produce cardiac glycosides? What is the interpretation from this phylogeny?
>>In the revised manuscript, we include the identified *Digitalis*, *Erysimum cheiranthoides*, and *Calotropis procera* genes in the CYP87A phylogeny shown in Figure 9B. This phylogeny identified a likely CYP87A homolog in *A. curassavica* based on the known functional protein from *Calotropis procera.*
>>Since cardiac glycoside biosynthesis evolved independently in the plant families containing the genera *Digitalis*, *Erysimum*, and *Asclepias*, the CYP87A proteins from these species are not likely to be more similar to one another than to genes from non-cardiac glycoside-producing plant species. By contrast, *Calotropis* and *Asclepias* are both in the Apocynaceae and are likely to share a common evolutionary origin of cardiac glycoside biosynthesis.

Figure S5 does not support the authors claim that “there is likely a homolog in A. curassavica (Figures S4 and S5).
>>We have changed this section of the manuscript to state that *Asclepias curassavica* genome encodes a CYP87A protein with 91% sequence identity to a protein from *Calotropis procera* that catalyzes the first step of cardiac glycoside biosynthesis.

Table S3: color scheme used is not clear. At one instance, it was used to show cytochrome 450 and in another instance, it was shown to represent for example, jasmonic acid biosynthesis. Use either metabolic/cellular process specific color scheme or genes specific color scheme.
>>We have added a legend to the top of Table S3 to explain the rationale for the color scheme that was used.

I am quite surprised that Authors did not emphasize on the expression pattern of known genes in cardiac glycoside biosynthesis. CYP87A4, 3bhsd, P5BR?? No mention about how many gene copies or isoforms exist for these genes? Their expression patterns across different tissues?
>>We have investigated the expression of the *Asclepias curassavica CYP87A* gene that is most similar to the known *Calotropis procera CYP87A* gene that is involved in cardiac glycoside biosynthesis. In the revised manuscript (Figure 9C), we include information about the expression of this gene in different tissue types.

>>P5BR genes have been investigated in *Digitalis* and *Erysimum*, which accumulate cardiac glycosides with the 5-beta configuration. By contrast, *Asclepias curassavica* makes 5-alpha cardiac glycosides. It is very unlikely that a steroid 5-beta reductase plays a prominent role in *A. curassavica* cardiac glycoside biosynthesis. Therefore, we did not look for expression of predicted P5BR genes in *A. curassavica*.

>>As in most plant genomes, there are predicted 3bhsd genes in *Asclepias curassavica.* However, so far there is no evidence that any 3bhsd enzymes are involved in *A. curassavica* cardiac glycoside biosynthesis. Based on the gene sequence alone it is not possible to predict whether any predicted *A. curassavica* 3bhsd genes are involved in cardiac glycoside biosynthesis or other plant steroid metabolism. Therefore, we feel that it is premature to emphasize the expression of such genes.

Again no metabolic data shown for any tissues. This is useful information to describe in the manuscript for a milkweed category plant, which is ecological model and substantial producer of cardiac glycosides

>>This manuscript presents the genome of *A. curassavica*. We did not measure metabolite content. We agree that it would be useful to measure cardiac glycoside content in different *A. curassavica* tissue types and compare the metabolite abundance to the gene expression data. As the seeds of our inbred *A. curassavica* isolate are publicly available from the USDA-ARS Ornamental Plant Germplasm Center (accession number OPGC 7586), any scientist who wants to conduct such measurements can do so.

Reviewer #2:

The authors present a high quality genome assembly along with associated tissue specific transcriptomes for Asclepias curassavica. The assembly appears to be highly complete ("chromosome level") based on comparison with existing genomic resources in Asclepias as well as BUSCO scores. RNA seq data was generated for plants with and without methyl jasmonate treatment and tested for differential expression of transcripts relative to treatment and tissue type. This paper is well written, and all of the methods are well established. The assembly and associated transcriptomic data represent a significant resource for future studies on cardiac glycoside biosynthesis and evolutionary biology of Apocyanceae. I am happy to recommend this paper for publication pending a couple of clarifications.

First, not being very familiar with cardiac glycoside biosynthesis/secondary metabolism, I'm not sure I

understand the significance of treatment with methyl jasmonate. A brief statement in the introduction explaining/justifying this treatment in relation to the stated interest in cardiac glycoside biosynthesis would help make this paper of broader interest.
>>In many plant species, the production of defensive metabolites is regulated by a jasmonic acid-dependent pathway. Thus, for a better annotation of genes that might be involved in plant defense, we measured gene expression after treatment with methyl jasmonate. The rationale for this is included in the revised manuscript.

In the results you list several genes/gene families (e.g. GGT1, GGPS, AMT1-2) that are up/down regulated across all tissues in response to MeJa treatment. However, you don't state why these specific genes are significant relative to the many other genes that are found to be differentially expressed across all tissue types. Why mention these specific genes?
>>In the revised manuscript, we provide a better explanation for why these genes were chosen as examples for altered expression in response to methyl jasmonate treatment. Some genes that were previously mentioned have been deleted because we did not consider them to be very relevant.

The wide range of genome size estimates is a bit odd, though not entirely surprising due to the limitations of flow cytometry and kmer analysis. Is there any specific explanation for this in Asclepias? Given the high sequencing depth with long and short reads, scaffolding with HiC, and synteny there is little question that this assembly is highly complete (but also see my question about the comparison with the A. syriaca genome above). However, this stood out to me as something that might merit more consideration.
>>There are a variety of limitations to estimating genome size with flow cytometry and kmer analysis. This is the most likely cause of the discrepancy in the size estimates. The assembled genome sequence that we present here is likely to be a more accurate estimate of the actual *A. curassavica* genome size.

**Response to reviewer Round 2:**

>>In response to comments from Reviewer 3, we have made changes to improve our manuscript. The specific changes that we made are detailed below. Additions to the text are highlighted in red in the "tracked changes" version of the resubmitted manuscript.

-------------------------------------------------------------------------- Reviewer comments:

Reviewer #2:

The authors have addressed all comments and questions about the manuscript to my satisfaction. I am happy to recommend it for publication.

Reviewer #3:

The authors worked on Asclepias curassavica, a tropical milkweed's de novo genome assembly, annotation, and tissue-specific transcriptomes of treated (with methyl jasmonate) and control to induce biotic defense pathways known to this plant. Although the technical methods (sequencing, assembly, annotation, synteny, gene-centric phylogeny, and DEG experiment and analyses) were scientifically sound, there were a few places where alternative computational methods could have clarified the inconsistencies.

Genome assembly and scaffolding review: For genome size discrepancies between flow cytometry (639Mbp), k-mer frequency (161Mbp) and assembly (241Mbp), one could do a K-mer frequency analysis of different sizes (other than k= 21) to verify the large heterozygous peak and heterozygosity of 0.2%. Also, demonstrating genome assemblers yielding a similar assembly size may validate the assembly size by MaSuRCA. ****The drastic genome size differences are a serious discrepancy in my opinion, so a varying kmer frequency analysis is a valid suggestion to incorporate. Also, the Lewis paper describes the 2C genome size, while the genome sizes reported here may be 1C.

>>Yes, we are describing 1C genome size, whereas the Lewis paper describes 2C genome size. We do not have a good explanation why our genome size estimate is smaller than that of Lewis et al. However, genome sequencing and assembly is almost certainly a more accurate measure of genome size than flow cytometry.

>>As suggested, we performed additional K-mer analyses, with lengths ranging from 19 to 29 (Figure S1), which showed that the 161 Mbp haploid genome size estimate is repeatable based on these data.

>>Given the 97.9% BUSCO score and 0.9% gene duplication, we are confident that the A. curassavica coding regions are well-represented in our MaSurCA assembly and scaffolding approach. Using a different assembly program is unlikely to change this result.

Since there are multiple mappings of the contact Hi-C map, stating the raw pre- and post-scaffolded assembly metrics, including how many gaps were filled vs joined creating the 233Mbp genome assembly (97% of 241Mbp contig assembly length) and if any of the 851 contigs unscaffolded would support the map. Are there any telomeric and/or centromeric sequences present in this assembly to show validity of the scaffolding (since)

>>Before scaffolding: Total assembly length = 241 Mbp and number of sequences = 851. After scaffolding and removal of duplicates (haplotigs): Total assembly length = 197 Mbp and number of contigs = 619. A more complete description of the assembly metrics, before and after scaffolding, has been added to Table S1.

>> We tried telomere identification tools and could not reliably identify telomeres or centromeres in the assembly.

Clearer synteny/gaps between 317Mbp of A. syriaca and the 241Mbp presented A. curassavica genome; i.e a large contracted area between the genomes or is the contracted areas dispersed evenly along the chromosomes

>>The contracted areas are dispersed in the genome. Contracted areas of the A. curassavica genome relative to A. syriaca are generally in regions of the A. curassavica genome that contain noncoding and repetitive regions. This information has been added to the manuscript text.

Figure 3: some axes have scale 0 to ~25Mbp and some do not, which make it hard for the reader to gauge synteny between the two genomes. It is puzzling that chromosome 1 seems to be contracted in the middle compared to the reference genome?

>> We have added additional marks at 5 Mbp intervals to designate chromosome size in the Circos plot in Figure 3.

Figure S3: Feature density along the Asclepias curassavica pseudomolecules. What do the colors mean? Seems bizarre that there is high gene density in the middle of chromosome 1 (usually gene density is located at the distal arms of a chromosome like it in in chromosome 1 of A. syriaca)?

>> Figure S3: Feature density along the Asclepias curassavica pseudomolecules. Track A = gene density, track B = repeat density. Darker shading represents regions with greater feature density. This information has been added to the legend for Figure S3.

Gene and expression analysis review: For the CYP87A gene family, are there any contractions or expansions in this gene family (since the CAFÉ analysis was done) **** No additional experiments are needed, only interpretation of results if available.

>> No, we do not see any significant expansions or contractions of this gene family along any of the lineages we tested. This observation is mentioned in the revised manuscript.

SMO and SS1 were pointed out to be involved in cardiac glycoside biosynthesis, however SMO seems to be downregulated in all tissues and SS1 is slightly upregulated across all tissues. Is there a hypothesis on why the biosynthetic genes are not upregulated in any tissue?

>>The two genes referenced by Reviewer 3, SMO (squalene monooxygenase) and SS1 (squalene synthase 1), are well-known genes encoding enzymes involved in plant terpene biosynthesis. As such, they provide precursors to many areas of plant metabolism, not only cardiac glycosides. Therefore, they may not be specifically upregulated by jasmonate elicitation.

I am not familiar with the pathway of interest, however, a clearer understanding of why certain genes of this pathway was looked at but not others could help readers also not familiar with the pathway (i.e. https://www.nature.com/articles/srep34464)

>>So far, CYP87A is the only verified Apocynaceae gene involved in cardiac glycoside biosynthesis (Kunert et al, 2023; research conducted with Calotropis procera). Therefore, we have presented expression of the Asclepias curassavica homolog of this gene in Figure 9C.

The research by Pandey et al (2016), which is referenced by Reviewer 3, involves C. procera transcriptome sequencing and identification of 336 genes potentially involved in cardiac glycoside biosynthesis. In the highly unlikely event that 100% of the C. procera cardiac glycoside biosynthesis

genes are in this 336-gene set, they would still constitute a small fraction of these 336 identified genes. Therefore, at this point, it would not be very productive to evaluate the expression patterns of the A. curassavica homologs of these 336 genes.

Miscellaneous:

Bud and flower are two of the tissue types, could you place add a development time-scale to this?

>>There is no defined developmental time scale for Asclepias curassavica. In our experience, the rate of flower development in A. curassavica depends on temperature and other unknown factors. Figure 1 shows that individual A. curassavica flower clusters can have both unopened buds and opened flowers.
